# Waking up quiescent neural stem cells: Molecular mechanisms and implications in neurodevelopmental disorders

**DOI:** 10.1371/journal.pgen.1008653

**Published:** 2020-04-23

**Authors:** Wei Yung Ding, Jiawen Huang, Hongyan Wang

**Affiliations:** 1 Neuroscience & Behavioural Disorders Programme, Duke-NUS Medical School, Singapore; 2 Department of Physiology, Yong Loo Lin School of Medicine, National University of Singapore, Singapore; 3 NUS Graduate School for Integrative Sciences and Engineering, National University of Singapore, Singapore; HudsonAlpha Institute for Biotechnology, UNITED STATES

## Abstract

Neural stem cells (NSCs) are crucial for development, regeneration, and repair of the nervous system. Most NSCs in mammalian adult brains are quiescent, but in response to extrinsic stimuli, they can exit from quiescence and become reactivated to give rise to new neurons. The delicate balance between NSC quiescence and activation is important for adult neurogenesis and NSC maintenance. However, how NSCs transit between quiescence and activation remains largely elusive. Here, we discuss our current understanding of the molecular mechanisms underlying the reactivation of quiescent NSCs. We review recent advances on signaling pathways originated from the NSC niche and their crosstalk in regulating NSC reactivation. We also highlight new intrinsic paradigms that control NSC reactivation in *Drosophila* and mammalian systems. We also discuss emerging evidence on modeling human neurodevelopmental disorders using NSCs.

## Introduction

**Fig 1:**
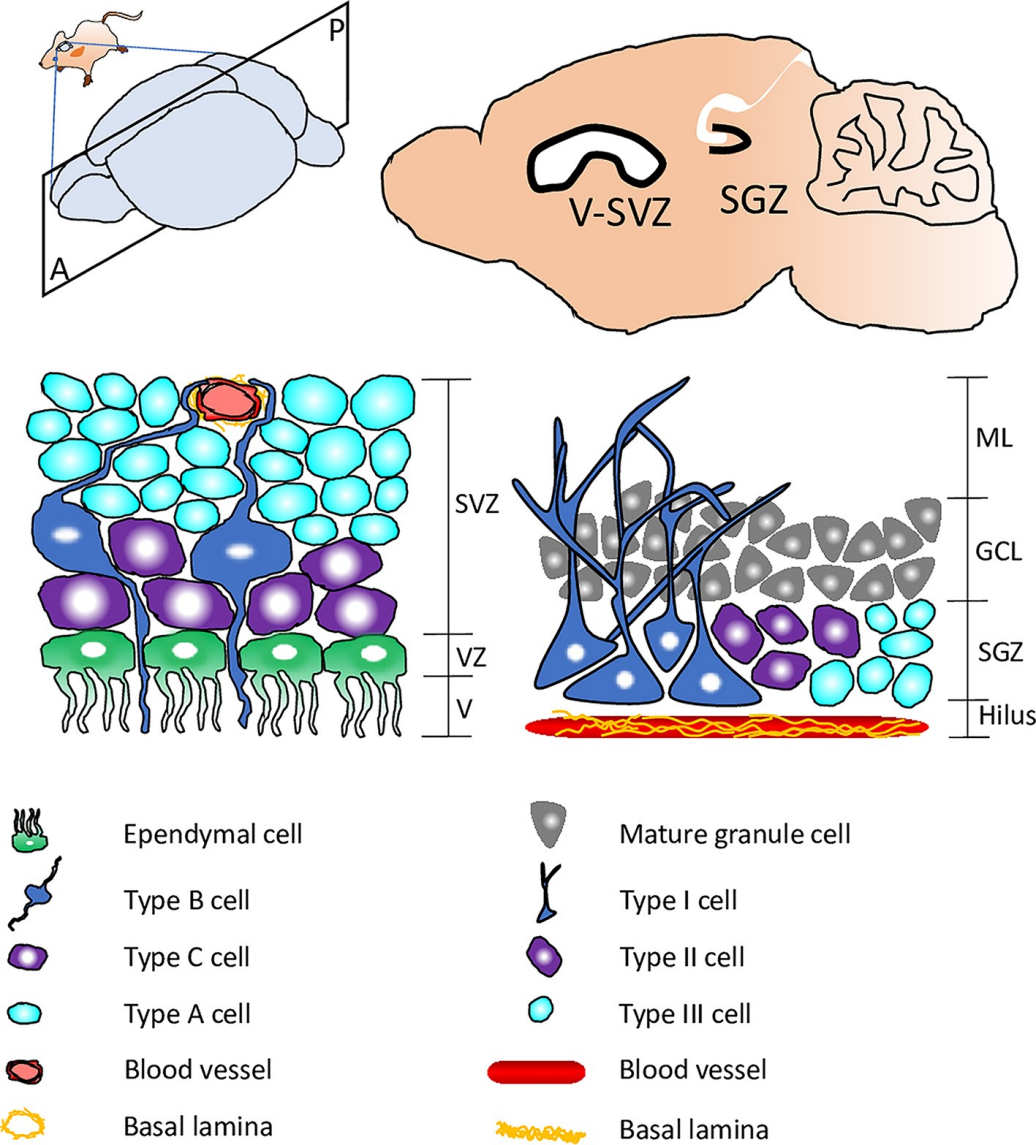
Schematic representation showing neurogenic niches within the mammalian adult brain. Top: a sagittal section of the mouse brain with neurogenic niches SGZ and V–SVZ highlighted. Bottom: schematics showing quiescent NSCs (type B in SVZ; type I in SGZ) and their surrounding cellular and molecular components within the V–SVZ (left) and SGZ (right). GCL, granule cell layer; ML, molecular layer; NSC, neural stem cell; SGZ, subgranular zone; SVZ, subventricular zone; V, ventricular space; VZ, ventricular zone.

The ability of stem cells to switch between quiescence and proliferation is crucial for tissue homeostasis and regeneration. Most neural stem cells (NSCs) in the mammalian adult brain exist in quiescence, a mitotic-dormant state, without undergoing proliferation or differentiation [1]. In response to physiological stimuli such as the presence of nutrients and physical exercise, quiescent NSCs can exit from quiescence and become reactivated to generate new neurons [2]. Conversely, stress, anxiety, and old age reduce the proliferation capability of NSCs [3]. Failure in NSC reactivation is thought to result in cognitive decline during old age [4]. In the mammalian adult brain, radial glial cells (type B) are NSCs that reside within the ventricular–subventricular zone (V–SVZ)/subependymal zone (SEZ) in the walls of the lateral ventricles, while radial glial cells (type I) are NSCs located in the subgranular zone (SGZ) of the hippocampal dentate gyrus (Fig 1) [5, 6].

**Fig 2:**
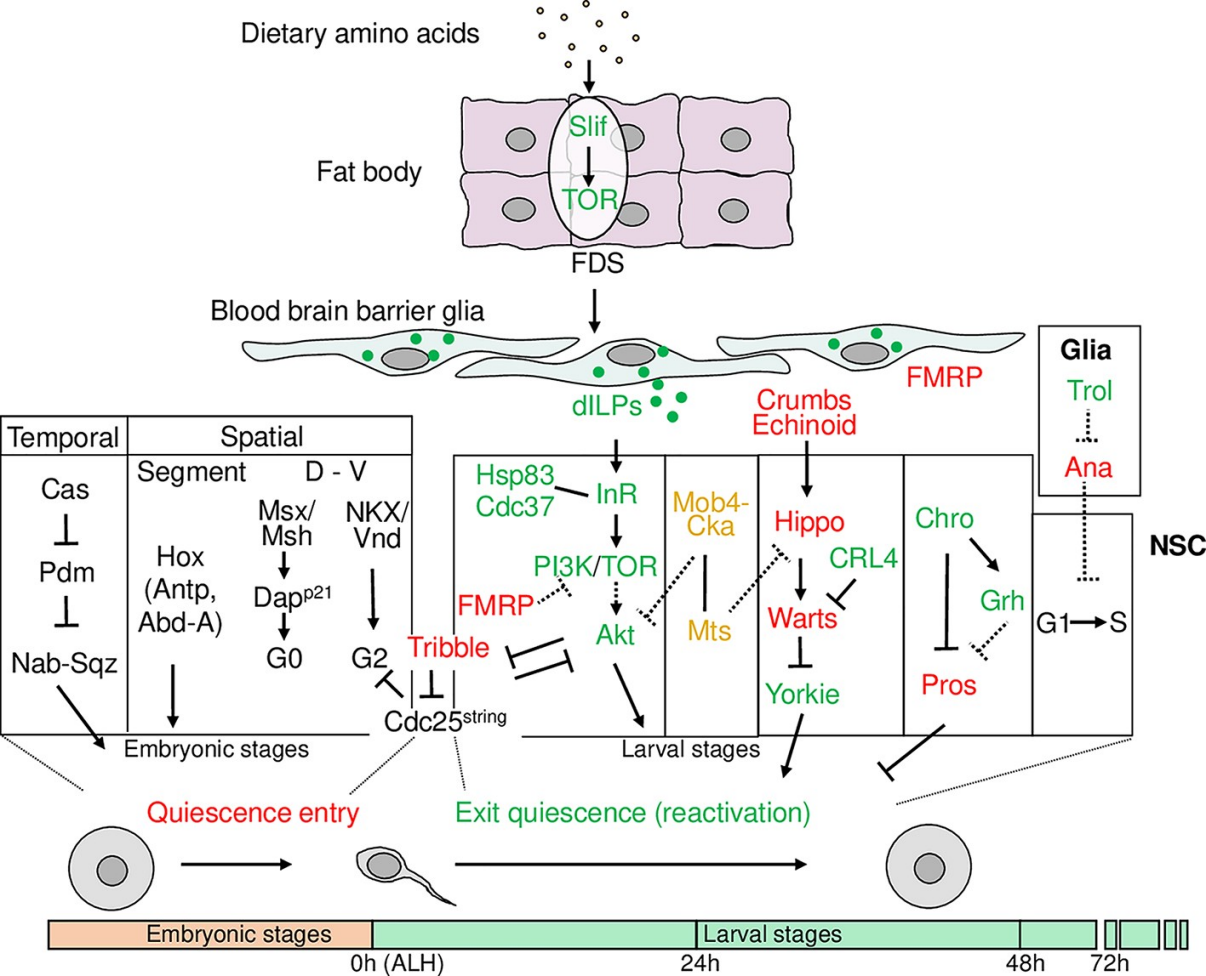
Schematic representation showing various factors within *Drosophila* fat body, BBB glia, and NSCs that regulate *Drosophila* NSC quiescence entry and reactivation. Factors promoting NSC reactivation are in green, while factors maintaining NSC quiescence or preventing reactivation are in red. Abd-A, Abdominal-A; ALH, after larval hatching; Ana, Anachronism; Antp, Antennapedia; BBB, blood–brain barrier; Cas, Caster; Cdc37, Cell division cycle 37; Chro, Chromator; Cka, Connector of kinase to AP-1; CRL4, Cullin-RING ligase 4; Dap^p21^, Dacapo (ortholog of p21CIP/p27KIP1/p57KIP2 family); dILPs, insulin/IGF-like peptides; D–V, dorsal to ventral; FDS, fat-body–derived signal; FMRP, Fragile X mental retardation protein; Grh, Grainy head; Hsp83, Heat shock protein 83; InR, Insulin receptor; Mob4, Monopolar spindle-one-binder family member 4; Msx/Msh, Muscle segment homeobox (ortholog of MSX1/2/3); Mts, Microtubule star; Nab, NGFI-A-binding protein; NKX/Vnd, Ventral nervous system defective (ortholog of NKX family); NSC, neural stem cell; Pdm, Pou-domain proteins Pdm1 and 2; PI3K, Phosphatidylinositol 3-kinase; Pros, Prospero; Slif, Slimfast; Sqz, Squeeze; TOR, Target-of-Rapamycin; Trol, Terribly reduced optic lobes.

NSCs in invertebrates such as *Drosophila melanogaster* also switch between a reversible transition between quiescence and reactivation [7–10]. *Drosophila* NSCs, also known as neuroblasts, enter quiescence for about 24 hours between embryogenic and postembryonic neurogenesis [7–10] (Fig 2). Because embryonic NSCs shrink their cell size following each cell division, by the end of the embryonic stage, the diameter of NSCs is decreased from approximately 10–14 μm to approximately 3–4 μm [7, 8]. Most NSCs in the abdominal regions of the ventral nerve cord (VNC) undergo apoptosis [11], while NSCs in the brain hemispheres and the thoracic VNC enter quiescence and subsequently exit quiescence during larval stages [8, 12, 13]. When larval NSCs exit quiescence, they undergo cell growth to reach the cell diameter of about approximately 7 μm before their first cell division in larval stages [14, 15].

*Drosophila* larval NSCs exit quiescence (reactivate) in response to feeding upon larval hatching [8, 12, 13]. The crucial dietary components for NSC reactivation are amino acids, but not nucleotide precursors, lipids, or vitamins [14]. However, none of the 11 essential amino acids alone in the food is sufficient for NSC reactivation, underscoring the importance of protein synthesis [14]. The signaling relay from the presence of dietary amino acids to the brain is controlled by an endocrine organ named the fat body, a functional equivalent of the mammalian liver and white fat [14, 16, 17]. The fat body senses circulating amino acids by the cationic amino-acid transporter Slimfast (Slif), leading to the activation of the Target-of-Rapamycin (TOR) pathway, which induces an unknown fat-body–derived signal (FDS) [16, 18]. The FDS is thought to reach the brain, stimulating NSC reactivation [14] (Fig 2). While extrinsic niche-derived cues allow NSCs to reactivate in respond to changes in the external environment such as the presence of nutrition, exercise, drug administration, or injury, intrinsic mechanisms represent another facet of control that is dependent on nuclear factors and cell-cycle regulators within NSCs during their reactivation.

## Signaling integration in the CNS barriers regulates the activation of NSCs

**Fig 3:**
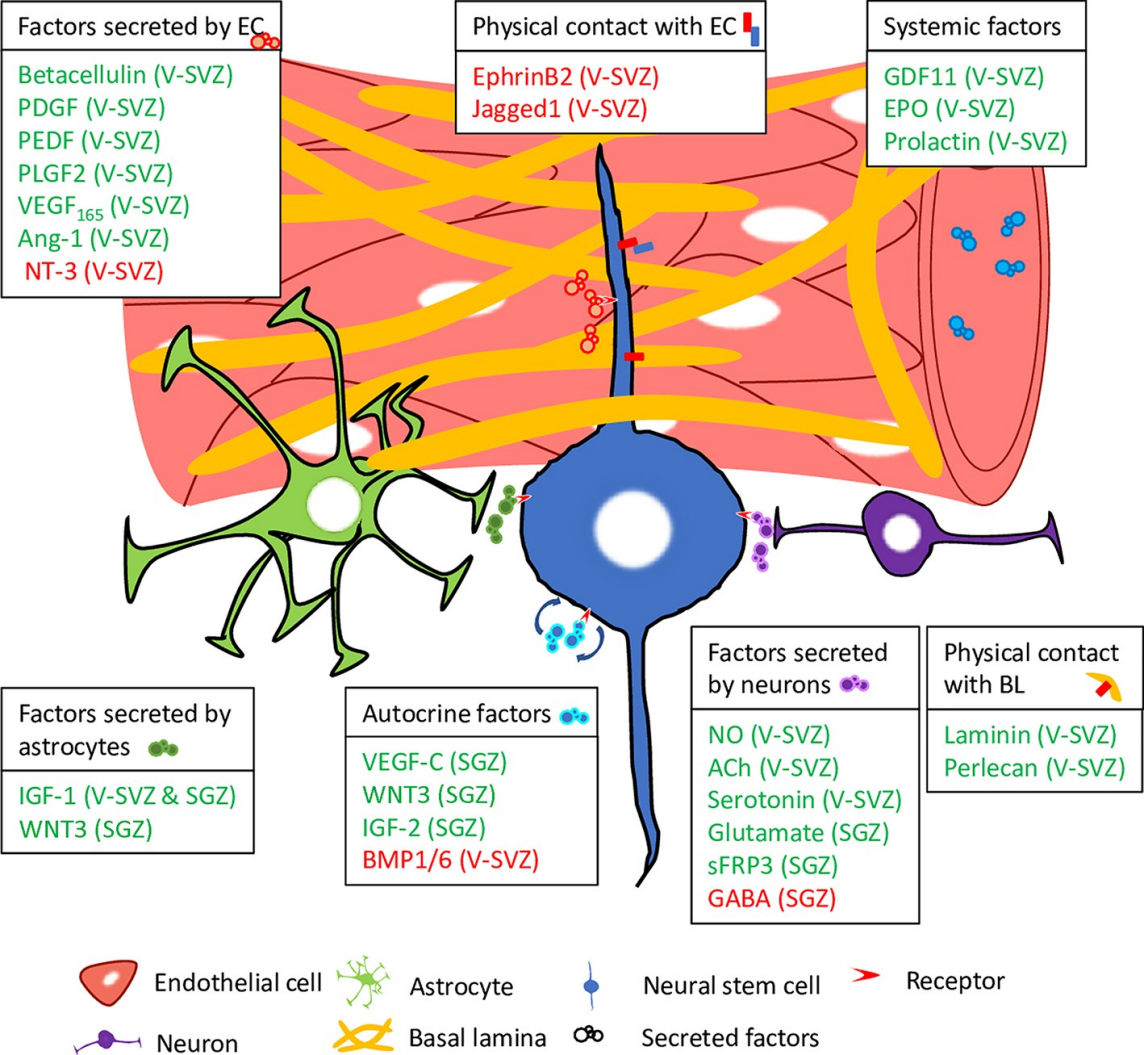
Schematic representation showing various factors within the neurogenic niche and from systemic circulation, as well as physical contacts with the microenvironment that regulate the balance between quiescence and reactivation of murine NSCs. Factors highlighted in green promote reactivation, while factors highlighted in red promote quiescence. Ach, acetylcholine; Ang-1, angiopoietin-1; BL, basal lamina; BMP1/6, bone morphogenetic protein 1/6; EC, endothelial cell; EPO, erythropoietin; GABA, gamma aminobutyric acid; GDF11, growth differentiation factor 11; IGF-1/2, insulin-like growth factor-1/2; NO, nitrous oxide; NT-3, neurotrophin 3; PDGF, platelet-derived growth factor; PEDF, pigment epithelium-derived growth factor; PLGF2, placenta-derived growth factor 2; sFRP3, secreted frizzled-related protein 3; SGZ, subgranular zone; VEGF-C, vascular endothelial growth factor-C; V–SVZ, ventricular–subventricular zone; WNT3, Wnt family member 3.

The blood–brain barrier (BBB) forms an insulation barrier to restrict free crossing of substances from the blood and protects the central nervous system (CNS) from toxins, inflammation, and pathogens while providing a microenvironment for neuroglia signaling [19] (Fig 3). The integrity of the mammalian BBB is primarily attributed to CNS endothelial cells that vascularize the brain [20]. These endothelial cells are connected by specialized intercellular tight junctions that have an important barrier function: to restrict paracellular permeability [21]. This permeability may be influenced by calcium oscillations of CNS endothelial cells [22]. In addition to their function as a barrier, CNS endothelial cells also supply the brain with essential nutrients by producing nutrient transporters such as glucose carrier, amino-acid carriers, and major facilitator domain containing 2A (Mfsd2a), a lysolipid transporter for docosahexaenoic acid (DHA) [23–25]. Mutations in human *MFSD2A* result in severe microcephaly syndrome, a neurodevelopmental disorder [26, 27]. Endothelial cells in the V–SVZ secrete factors that have an opposing effect in activating or maintaining quiescence of NSCs [28, 29]. To activate NSCs, betacellulin acts on epidermal growth factor receptor (EGFR), activating the extracellular signal-regulated kinase (ERK)/AKT, also known as protein kinase B (PKB), pathway to enter a proliferative stage [28]. On the other hand, neurotrophin 3 (NT-3) up-regulates endothelial isoform of nitrous oxide synthase (eNOS) that promotes NSC quiescence in a nitrous oxide (NO)-dependent manner [29]. In vitro study has, however, demonstrated a dose-dependent effect of NO in the balance of NSC quiescence–activation, with low concentration resulting in an increase in cell proliferation, while high concentration resulted in a decrease in cell proliferation [30]. Further investigations into the mode of NO regulation within the BBB niche will shed light on the dynamic regulation of NSC activation under homeostatic conditions and in response to external insult.

Systemic signals delivered via the vasculature to the BBB niche have been implicated in the regulation of NSC activation. Using a mouse heterochronic parabiosis model by surgically joining pairs of animals, systemic factors derived from young mice, specifically identified to include growth differentiation factor 11 (GDF11), are shown to drive vascular remodeling and activation of NSC proliferation in the V–SVZ of aged mice [31]. Conversely, systemic factors derived from aged mice have an attenuating effect on NSC proliferation in the SGZ [32, 33]. In the V–SVZ, hormones such as erythropoietin and prolactin have positive effects in activating the proliferation program in quiescent NSCs [34, 35]. However, prolactin has a negligible effect on NSCs residing in the SGZ, suggesting a differential effect of hormones and possibly other soluble factors that depends on spatial cues and niche characteristics [34].

Besides endothelial cells, the brain microenvironment also contains brain pericytes, neurons, and astrocytic glia that influence barrier properties [36]. Astrocytes that extend cellular processes ensheathe the blood vessels by contacting and surrounding CNS endothelial cells through the endfeet of their basal processes [19]. Astrocytes regulate the permeability of the BBB and secrete factors such as transforming growth factor beta (TGF-β), glial-cell–derived neurotrophic factor (GDNF), and basic fibroblast growth factor (FGF) that regulate BBB development [25].

The *Drosophila* brain is separated from the blood-like hemolymph by the functional analogue of BBB [37]. The *Drosophila* BBB in larval stages is composed of 2 types of surface glia named perineural glia (PG) and subperineural glia (SPG) [38, 39]. The PG characterized with a stellate appearance are located at the outer layer, while the SPG with sheet-like morphology are located immediately beneath the PG [40–42]. The SPG are the major BBB layer because they form septate junctions at the lateral borders between the SPG cells. The BBB glia provide an important niche for the regulation of NSC quiescence and reactivation via various signaling pathways.

### The insulin pathway promotes NSC reactivation

In response to nutritional input, insulin/insulin-like growth factor (IGF) signaling (IIS) controls growth, metabolism, and longevity [43]. The function of IIS in growth is evolutionarily conserved in *Drosophila*, in which there are a single insulin/IGF receptor (dInR) and 8 insulin/IGF-like peptides (dILPs 1–8) [44, 45]. There are at least 2 source of dILPs in the *Drosophila* larval brain, the specialized neurosecretory cells named insulin-producing cells (IPCs) and a set of surface glia overlying the NSCs [15, 44]. Functioning analogous to β cells of the vertebrate endocrine pancreas, IPCs produce and secrete dILP1, 2, 3, and 5 into the hemolymph and act systemically to regulate larval growth and lipid metabolism [46, 47]. By contrast, surface glia in the larval brain are dispensable for systemic growth but essential for NSC reactivation [15, 18]. During NSC reactivation in the *Drosophila* VNC, the production of dILP2 and dILP6 increases [15]. The dILP2 and dILP6 are found to be produced in a subset of PG glia that have a stellate morphology and are located between the NSCs and basement membrane [15, 18]. dILP3 expression was found in some glia and neurons in the CNS in the early second-instar stage [18], suggesting that dILP3 may play a role at later developmental stages following NSC reactivation. Overexpression of dILP2 or dILP6 in glia is sufficient for NSC reactivation in the absence of dietary amino acids without apparently altering larval growth [15, 18]. On the contrary, none of the dILPs 1–7, upon overexpression in IPCs, could reactivate NSCs under nutrient restriction conditions [18]. Therefore, *Drosophila* NSCs respond to a local source of dILPs from glial cells, but not the systemic source from IPCs, to exit quiescence. Presumably, the mitogen from the fat body stimulates the glial cells to produce and secrete dILP2 and dILP6 [15, 18, 48]. The identity of this fat-body–derived mitogen—growth factors, hormones, or signaling molecules—remains elusive. The dILPs secreted from the glial cells act locally by directly activating the insulin receptor (InR)/phosphatidylinositol 3-kinase (PI3K)/Akt pathway as well as the TOR pathway in underlying NSCs [15, 18] (Fig 2). As a result, protein biosynthesis begins, and NSCs re-enter the cell cycle through inhibition of the Forkhead box O (FOXO) transcription factor [15, 18].

NSC reactivation occurs relatively synchronously, in about 24 hours, in all neurogenic regions of the *Drosophila* CNS [15]. This is achieved by the function of gap junctions in the BBB glia that couple metabolic signal with synchronized calcium pulses and insulin secretion [49]. The gap junction is a transmembrane channel formed by docking of connexin hexamers from adjacent cells [50]. Gap junction proteins are required in the BBB glia for the secretion of dILP6 and subsequent coordinated calcium oscillations of SPG [49]. The inositol-triphosphate (IP3) binds to its receptor Ins3PR, a calcium channel in the endoplasmic reticulum (ER), and releases calcium from intracellular stores in glial cells to trigger NSC reactivation [49]. It is important to note that depletion of gap junction proteins does not cause the leakage of BBB because the septate junctions of the BBB glia appear to be intact [49].

Analogous to *Drosophila* BBB glia, in the mammalian adult hippocampus, astrocytic glia function as a niche to induce neurogenesis by promoting proliferation of NSCs and neuronal fate commitment [51]. This was first demonstrated by coculturing of adult NSCs with primary hippocampal astrocytes, which is sufficient to promote neurogenesis [51]. Astrocytes produce IGF-1, which promotes NSC proliferation in mammalian adult brains [52]. IGFs, namely InR, IGF-1 receptor (IGF-1R), and IGF-2R, are also abundantly expressed in the mammalian brain [53, 54]. IGF-1 is expressed in astrocytes, neurons, and NSCs in the hippocampus and the V–SVZ, and its expression in the brain is much higher than in systemic circulation during neurogenesis [55–58]. Locally overexpressed or directly infused IGF-1 can trigger NSC proliferation without leading to an increase of IGF-1 level in the circulation [59, 60], suggesting that locally expressed (paracrine or autocrine) IGF-1 is crucial for regulating NSC proliferation. This mitogenic role of IGF-1 leads to the activation of mammalian target of rapamycin complex 1 (mTORC1) and inhibition of FOXO via the PI3K/Akt pathway [61–64]. Both IGF-1 and the PI3K/Akt pathway promote cell-cycle progression [65, 66]. IGF-2 also promotes the proliferation of NSCs via Akt signaling, and it is highly expressed in NSCs in the hippocampal dentate gyrus [67]. Thus, the InR/PI3K/Akt pathway appears to be a common theme in promoting NSC reactivation in both flies and mammalian NSCs.

Dysregulation of critical components in the PI3K/Akt pathway has been implicated in neurodevelopmental disorders [68]. Three common mutations of IGF-1R and deletion of the chromosome region containing *AKT3* have been identified in patients with primary microcephaly [69, 70], suggesting that IGF-1R mutations and *AKT3* deletion may contribute to this neurodevelopmental disorder. Removing downstream effectors of the PI3K/Akt pathway in vivo, e.g., phosphoinositide-dependent kinase 1 (Pdk1), mTOR, and raptor, has also been found to cause microcephaly [71–75]. Conversely, mutations activating *PI3KCA* and *AKT3*, the predominant AKT isoform in mouse brain cortex and hippocampus, have been linked to clinical manifestations of a spectrum of enlarged brain malformations, e.g., macrocephaly, dysplastic megalencephaly, and hemimegalencephaly [72, 76, 77]. Similarly, the loss of phosphatase and tensin homolog (PTEN), a PI3K antagonist, results in increased cell proliferation and reduced cell death, which contributes to macrocephaly [78]. Subsequent study demonstrates that conditional deletion of PTEN up-regulates the reactivation of NSCs in the SGZ [79].

### Other signaling pathways and proteins from glia control NSC proliferation

One of the earliest evidence on the involvement of glia in NSC reactivation was from a study on *Drosophila* DE-Cadherin, a cell adhesion molecule, which acts in glia cells to promote the proliferation of NSCs [80]. Besides the InR/PI3K/Akt pathway, several major evolutionarily conserved signaling cascades also regulate NSC reactivation. The TGF-β/BMP (bone morphogenetic protein) pathway plays crucial roles during various cellular processes such as cell growth and differentiation. The BMP signaling pathway promotes NSC proliferation in *Drosophila* because Glass bottom boat (Gbb), a BMP homolog that is expressed in NSCs, acts as an autocrine proliferation factor in NSCs [81]. Dally-like (Dlp), a heparan sulfate proteoglycan protein on the cell surface and in the extracellular matrix, functions as a coreceptor for Gbb in PG to promote NSC proliferation [81]. Interestingly, NSC-expressing Gbb also provides a paracrine signal for the survival of PG, suggesting that a bidirectional communication between NSCs and the BBB glia influences the development of both cell types [81]. In the mammalian adult brain, the BMP pathway blocks neurogenesis and directs glial differentiation of NSCs [82]. The BMP target inhibitor of differentiation 2 (ID2), its ligand BMP1, BMP6, and BMP receptor BMPR1B are all expressed in quiescent NSCs, suggesting that BMP regulates NSC proliferation in an autocrine manner [83]. BMP maintains NSC quiescence, as the loss of BMP signaling via selective ablation of upstream BMPR-IA receptor leads to a transient increase in NSC proliferation, followed by depletion of stem cell pool in the long term [84]. The inhibition effect on neurogenesis by the BMP pathway can be antagonized by Noggin through paracrine secretion by ependymal cells adjacent to the V–SVZ [82]. Similarly, Noggin expression is found in the adult dentate gyrus, antagonizing the BMP pathway to promote the proliferation of NSCs [85]. Interestingly, BMP signaling has a surprising divergent effect in promoting proliferation and quiescence in NSCs of *Drosophila* and adult mice, respectively. Nevertheless, because studies in *Drosophila* were carried out in third-instar larval brains, it remains to be determined whether BMP signaling is required for NSC reactivation in early stages and/or maintenance of NSC proliferation at later stages.

Another heparan sulfate proteoglycan protein named Terribly reduced optic lobes (Trol), the *Drosophila* perlecan homolog, is also required for G1/S transition during NSC reactivation [86, 87]. Trol is expressed in a subset of dorsal midline glial cells of the CNS [86], suggesting that it functions non-cell–autonomously for NSC proliferation. It is believed that Trol promotes NSC reactivation through antagonizing the NSC reactivation inhibitor Anachronism (Ana) [86] (Fig 2). In addition, Trol interacts with both FGF-2 and Hedgehog (HH) in larval protein extracts [88]. The low affinity binding between heparan sulfate proteoglycan proteins and FGFs is required for the binding of FGFs to their high affinity receptors [89]. Subsequent study in mice recapitulated the conserved role of perlecan in mediating FGF-2 signaling that promotes V–SVZ NSC proliferation [90]. In adult mammalian brains, the mitogens epidermal growth factor (EGF) and FGF-2 promote NSC proliferation [91, 92]. The activity of FGF-2 can be modulated by its low affinity receptor heparin, which either activates or inhibits the mitogenic activity of FGF-2 on NSCs, likely depending on the structure, composition, or expression level of heparin on the cell surface [93, 94].

The basal lamina, a layer of extracellular matrix (ECM) secreted by the CNS endothelial cells, provides an important molecular signature for adult mammalian NSCs to modulate its activation of proliferation program [95]. During development and postnatal stages, a failure in deposition of ECM proteins, including Laminin-α4, surrounding the vasculature leads to detachment of the critical linkage between endothelial cells and V–SVZ NSCs [96]. Compared to those at quiescent stage, activated NSCs in the V–SVZ have increased expression of certain ECM receptors such as laminin receptor α6β1-integrin and syndecan-1 [97–99]. The activation of NSCs in the V–SVZ are further enhanced by their ability to bind to laminin in the vascular niche via an up-regulation of EGFR and α6 integrin by stromal-derived factor 1 (SDF1) [100]. Further elucidations on the role of ECM and other BBB niche cells such as pericytes in modulating NSC activation may provide insights into generating improved regenerative therapeutics for brain trauma and cerebral ischemia.

*Drosophila* Hh signaling activates NSC division in a Trol-dependent manner [88]. Mammalian Sonic hedgehog (Shh) regulates adult NSC proliferation in rat hippocampus and the V–SVZ [101, 102]. Quiescent NSCs in the V–SVZ and SGZ respond to Shh and are able to self-renew and expand the NSC population for about 1 year in vivo [103]. In the mammalian adult hippocampal SGZ, *Wnt3* is expressed in astrocytes, and its overexpression is sufficient to increase neurogenesis by controlling the neuronal fate commitment and proliferation of neural precursor cells and neuroblasts, while inhibition of Wnt signaling reduces adult neurogenesis [104]. Disrupted In Schizophrenia 1 (DISC1), which is deficient in patients with schizophrenia, depression, and bipolar disorder, is expressed in adult mouse NSCs and regulates their proliferation [105]. DISC1 directly associates and inhibits glycogen synthase kinase 3 beta (GSK3β) activity, leading to a reduction of β-catenin phosphorylation and its stabilization [105]. In support of these findings in mouse NSCs, altered WNT signaling has been identified in human NSCs derived from human induced pluripotent stem cells from schizophrenia patients [106].

In summary, in both *Drosophila* and mammalian NSC niches, ECM plays an important role in regulating the balance of quiescence and proliferation. Not only does it serve as a physical support for NSC anchorage, it also acts as a depositing scaffold for various secreted factors by niche cells and from systemic circulation. Nevertheless, the components of ECM in both *Drosophila* and mammalian systems remain poorly defined, making in-depth characterization of NSC–ECM interaction difficult. Because mesenchymal stem cell quiescence–proliferation decision was shown to be dependent on substrate stiffness [107], further investigation on the mechanobiology of ECM in regulating NSC quiescence, i.e., elasticity, stiffness, microtopography, etc., is warranted.

### The inhibitory role of the CNS barrier in NSC reactivation

On the flip side, the *Drosophila* glia niche also provides inhibitory factors that maintain NSC quiescence. Glial cells secrete the glycoprotein Ana to prevent NSCs from entering S-phase, therefore maintaining NSC quiescence [108]. Glia are also required for the activation of the evolutionarily conserved Hippo pathway that keeps NSCs in quiescence [109]. First identified in *Drosophila*, the Hippo pathway plays a conserved role in tumorigenesis, organ development, and stem cell maintenance [110]. In the absence of dietary amino acids, 2 intercellular transmembrane proteins Crumbs and Echinoid are expressed in both NSCs and their glial cell niche [109]. This intercellular interaction of Crumbs and Echinoid activates the Hippo pathway composed of Tao-1, Hippo, Salvador, and large tumor suppressor (Lats)/Warts in *Drosophila* NSCs [109, 111–113]. This growth-repressive kinase cascade ultimately phosphorylates the transcriptional coactivator Yorkie, resulting in its cytoplasmic retention [109, 114]. In the presence of dietary amino acids, Echinoid was down-regulated mainly in glia over time, and Crumbs is lost in both glia and NSCs, leading to the inactivation of the Hippo pathway and, in turn, translocation of Yorkie into the nucleus to activate downstream targets such as *bantam* microRNA, ultimately triggering NSC reactivation [109]. Although loss of the Hippo pathway causes premature NSC reactivation on the fed condition, it is unable to overcome the requirement of dietary amino acids [109]. It is unknown how the nutritional status alters the expression of Crumbs and Echinoid in the brain. Possibly the protein turnover or the intracellular trafficking of Crumbs and Echinoid is controlled in response to nutrition.

Notch signaling has been implicated in the maintenance of mammalian NSC quiescence because Notch downstream effector Hes family bHLH transcription factor 1 (*HES1*) and *HES5* inhibit neuronal differentiation [115]. The physical contact maintained between NSCs in the V–SVZ and endothelial cells allows ligands ephrinB2 and Jagged1, which are expressed by endothelial cells, to trigger ephrine (Eph) and Notch signaling in the NSCs to maintain quiescence [116]. This was achieved synergistically by inhibiting proliferation through Eph signaling and blocking differentiation via Notch signaling. Inhibition of Notch signaling results in a transient increase in NSC proliferation, followed by stem-cell–pool depletion in the long term [117–119]. Notch ligand delta-like protein 1 (*Dll1*), which is expressed in activated NSCs and subsequently segregated into the daughter cell undergoing differentiation after asymmetric division, maintains quiescence of adjacent NSCs, suggesting a feedback loop for NSC maintenance between sister cells [120]. Patients with deleterious mutations in the *DLL1* gene are found to have developmental delay, intellectual disability, and brain malformations [121].

In a pioneer study conducted by Palmer and colleagues, they demonstrated that proliferating NSCs within the SGZ can be found close to angiogenic capillary tips, suggesting a possible role for angiogenic regulators in NSC activation [122]. A subsequent study on platelet-derived growth factor (PDGF) in the V–SVZ, a well-known angiogenic factor, in which its in vivo introduction led to formation of hyperplastic nodules containing highly proliferating NSCs, strongly suggests its role in activating NSC proliferation [123]. This was followed by the elucidation of pigment epithelium-derived factor (PEDF), angiopoietin-1 (Ang-1), and vascular endothelial growth factor 165 (VEGF_165_), all of which were found to be involved in activating NSC proliferation in the V–SVZ [124–126]. In addition, placenta-derived growth factor 2 (PLGF2), an important player in endothelial stimulation and pathological angiogenesis, activates NSCs in the V–SVZ niche by interacting with vascular endothelial growth factor 1 (VEGFR1) [127]. Similarly in the SGZ, VEGF-C interacts with VEGFR3, which activates quiescent NSCs through the ERK/AKT pathway [128]. It is interesting to note that while PDGF, Ang-1, VEGF, and PLGF2 are proangiogenic, PEDF is instead antiangiogenic. Such opposing regulations of angiogenesis that converge in activating NSCs suggest that dynamic neurogenesis occurs in both physiological and pathological situations. Future studies on the synergistic effect of these angiogenic regulators on NSC activation in conjunction with angiogenic sprouting or remodeling in the BBB niche are warranted.

### Remodeling of cortex glia during NSC reactivation

In the *Drosophila* larval CNS, each NSC and its progeny are individually surrounded by cortex glial membrane to form the NSC lineage within the chamber [129]. How can the BBB glial signals reach NSCs if NSC lineages are enclosed by the cortex glia? Speder and Brand showed that in early larval stages, NSCs are not covered by the cortex glial membrane, allowing direct contact between the BBB glia and NSCs [130]. Once NSCs reactivation is completed by 48 hours after larval hatching, the cortex glia chambers are closed at around the same time [130]. The development of cortex glial chambers is also dependent on nutrition—essential amino acids [130]. NSC reactivation drives the formation of cortex glial chambers in both fed and nutritional restriction conditions [130]. The intact cortex glial chambers are crucial for maintaining the survival of newborn neurons, but not NSC survival or proliferation [130].

### The role of neurons in NSC reactivation

Innervation of stem cell niches in the V–SVZ and SGZ by projections from proximal and distal neurons have distinct effect in regulating NSC reactivation. In the adult dentate gyrus, the cellular processes of NSCs wrap around the cell bodies of granule neurons and touch and/or ensheathe putative glutamatergic synapses likely formed between mossy cells (MCs), a major type of excitatory neurons, and mature granule cells [131]. Indeed, granule neurons release secreted frizzled-related protein 3 (sFRP3) to maintain NSC quiescence in the SGZ [132]. The differential activation of MCs regulates the balance of quiescence and reactivation of NSCs within the SGZ, in which the direct MC–NSC glutamatergic pathway favors reactivation and indirect MC–interneuron–NSC gamma aminobutyric acid (GABA)ergic pathway favors quiescence [133–135]. In the V–SVZ, a new population of choline acetyltransferase (ChAT)^+^ neurons release acetylcholine to stimulate NSC proliferation [136]. In addition, serotonergic axons originated from neurons in the raphe nuclei exert a positive effect on the proliferation of NSCs in the V–SVZ [137]. Nitrergic neurons located in close proximity to the adult V–SVZ regulate NSC proliferation in a negative manner [138]. In *Drosophila*, quiescent NSCs extend their primary cellular extension into the neuropil [8, 15], raising the intriguing possibility that neurons may also function as a niche to regulate *Drosophila* NSC reactivation.

## Intrinsic mechanisms controlling NSC reactivation

Intrinsic mechanisms in certain stem cell subpopulations could play a dominating role in regulating their behavior. Indeed, NSCs from different spatial niches within the V–SVZ reactivate to give rise to neurons that are phenotypically reminiscent of their site of origin even when transplanted heterotopically [139]. Such an intrinsic response is governed by regulators that are often transcription factors, epigenetic modifications, and cell-cycle regulators.

### Controlling NSC reactivation by regulators of the InR/PI3K/Akt, BMP, and Hippo pathways

With the discoveries on the roles of various signaling pathways in NSCs and their niche, recent studies have identified regulators of these signaling pathways that are critical for NSC quiescence and reactivation. Heat shock protein 83 (Hsp83), a Hsp90 family molecular chaperone, is an intrinsic regulator of the dInR pathway during NSC reactivation [140]. Hsp83, together with its cochaperone Cell division cycle 37 (Cdc37), facilitates the activation of dInR and promotes NSC reactivation intrinsically [140]. Hsp83 likely binds to dInR in NSCs in a near-native state poised for activation by binding of dILPs [140]. In the presence of dietary amino acids, the expression of *hsp83* is dramatically up-regulated, which serves as an additional mechanism for activation of the dInR pathway in NSCs in response to nutritional stimuli [140]. The interaction between Hsp90 and InR is conserved in mammalian systems. In human fibroblasts, Hsp90 promotes insulin signaling in mitogenesis through interaction with intracellular InR β subunit [141]. In mammals, the expression level of Hsp90 in the brain is the highest among all tissues [142]. Hsp90’s clients include α-synuclein in Parkinson's disease and tau in Alzheimer's disease, and therefore, it is heavily implicated in neurodegenerative diseases [143].

Fragile X mental retardation protein (FMRP) is an RNA-binding protein, and its deficiency causes Fragile X syndrome, the most common genetic form of intellectual disability (ID) and autism spectrum disorders (ASDs). *Drosophila* FMRP is expressed in both NSCs and glial cells, and it prevents NSC reactivation by inhibiting the InR/PI3K/Akt pathway in NSCs and an unknown mechanism in the glia [144, 145]. Like its *Drosophila* homolog, mammalian fragile X-related protein 2 (FXR2P) inhibits NSC proliferation in the adult hippocampus by up-regulation of BMP signaling [146]. Mammalian FMRP and FXR1P and FXR2P, 2 other proteins from the same family, play distinct regulatory roles in adult neurogenesis, including NSC proliferation, transition from NSCs to intermediate progenitor cells, and neuronal maturation [146–148].

The protein turnover of Lats/Warts, a core protein kinase in the Hippo pathway, is regulated by a Cullin-really interesting new gene (RING) ligase named CRL4^Mahjong^, an evolutionarily conserved E3 ubiquitin ligase composed of Cullin4 (Cul4), DNA damage-binding protein 1 (DDB1), regulator of cullins-1 (Roc1), and a substrate receptor named Mahjong [149]. Both DDB1 and Mahjong are up-regulated in reactivated NSCs compared with quiescent NSCs and are required for NSC reactivation [149]. Depletion of *ddb1* or *mahjong* in NSCs leads to delayed NSC reactivation and a microcephaly-like phenotype [149]. CRL4^Mahjong^ targets Warts for ubiquitination and degradation, therefore releasing Yorkie into the nucleus to trigger NSC reactivation [149]. The interaction between CRL4 and Warts/Lats is conserved because in human cancer cells, CRL4 E3 ligase activity is increased, leading to the ubiquitination and down-regulation of Lats1/2 [150].

The role of mammalian Hippo pathway and CRL4 complex in NSC reactivation is largely unknown. Upon BMP4-induced mouse adult NSC quiescence, WW and C2 containing domain 2 (WWC2) (Kibra homolog), Lats2 (Warts homolog), and Crumbs2 (Crumbs homolog) are up-regulated [151]. Whether Crumbs activates the hippo pathway to maintain NSC quiescence in mammalian adult brains remains to be determined. Rat Cul4B is highly expressed in mitotic NSCs and its knockdown arrests primary NSCs at G2/M transition [152]. Analogous to the microcephaly-like brains observed in *Drosophila ddb1* mutants, in the mouse developing brain, a CNS-specific depletion of DDB1 leads to decreased NSC proliferation and the formation of smaller brains [153]. In zebrafish, the CRL4 complex with a substrate receptor named cereblon (CRBN) controls NSC proliferation and brain size [154, 155]. Zebrafish *ddb1*- or *CRBN*-depleted embryos develop smaller brains with a reduction of the number of proliferating cells [154, 155]. Variants of human Cul4B are associated with neurodevelopmental disorders, including X-linked ID, mental retardation, and cortical malformations [156–159].

The opposing roles of the InR/PI3K/Akt and Hippo pathways are coordinated by members of the conserved striatin-interacting phosphatase and kinase (STRIPAK) complex [160]. STRIPAK members are found to have differential expression in quiescent and reactivating NSCs through a transcriptional profiling [160]. *microtubule star* (*mts*), the catalytic subunit of protein phosphatase 2A (PP2A), maintains NSC quiescence primarily by inactivating Akt [160]. Two other components of STRIPAK, named monopolar spindle-one-binder family member 4 (mob4) and connector of kinase to AP-1 (cka), promote NSC reactivation by facilitating the association between Mts and Hippo, presumably resulting in the dephosphorylation and inactivation of Hippo [160]. Therefore, the STRIPAK members first turn off the InR/PI3K/Akt pathway to maintain NSC quiescence and subsequently turn off the Hippo pathway to promote NSC reactivation. Interestingly, Cerebral cavernous malformation 3 (Ccm3), a STRIPAK component, is expressed in the CNS BBB and modulates the organization and function of the BBB [161, 162].

Adenomatous polyposis coli (APC) family proteins APC1 and APC2, negative regulators of the Wingless/Wnt pathway, play a redundant role in *Drosophila* larval NSC reactivation, but loss of both APC1 and APC2 did not seem to result in any accumulation of β-catenin (Armadillo) in NSCs [163]. Whether the Wingless pathway is involved during NSC quiescence and reactivation awaits further investigation.

### Transcriptional and epigenetic regulations of NSC reactivation

At the end of embryogenesis, exit of proliferation of *Drosophila* NSCs is controlled by combined functions between temporal transcriptional factors and spatial regulators such as Hox proteins [12] (Fig 2). Temporal transcriptional factors Pou-domain proteins Pdm1 and Pdm2 (Pdm) prevent NSC quiescence through down-regulation of Nab, as Nab normally induces NSC quiescence with its co-factor Squeeze [12]. Another temporal transcriptional factor, Castor, promotes quiescence by inhibiting Pdm [12] (Fig 2). Differential expression of Hox genes Antennapedia (Antp) and Abdominal-A (Abd-A) is responsible for the different timing of entry into quiescence in different segments [12].

Homeodomain transcription factor Prospero (Pros) is well-known for its role in neural differentiation by directly repressing progenitor and cell-cycle genes [164, 165]. Pros is also capable of driving proliferating NSCs into quiescence when transiently expressed in NSCs [166]. The levels of Pros in the nucleus distinguish *Drosophila* NSC fates: absence for self-renewal/proliferation, low for quiescence, and high for differentiation [166]. Pros is repressed by spindle matrix proteins composed of Chromator (Chro)/chromo domain protein interacting with Z4 (Chriz), Megator, and enhanced adult sensory threshold (East) that function intrinsically in NSCs to promote NSC reactivation [167] (Fig 2). Chro also promotes the expression of *grainy head*, which indirectly represses *pros* expression in NSCs [167]. Chro appears to function downstream of the InR/PI3K pathway during NSC reactivation, although it remains unknown whether Chro is a direct target of the InR/PI3K pathway [167].

Several transcription factors play counterbalancing roles in the regulation of mammalian NSC quiescence and reactivation [168–171]. Achaete-scute homolog 1/mammalian achaete scute homolog 1 (ASCL1/MASH1), a proneural basic helix–loop–helix transcription factor, promotes the activation of quiescent NSCs in both the adult V–SVZ and hippocampus [172]. The expression of *Ascl1* in NSCs can be induced by neurogenic stimuli or inactivation of the Notch signaling pathway [172]. Oscillatory or sustained expression of *Ascl1* regulated by its repressor HES1, a downstream effector of Notch signaling, determines whether NSCs commit to a renewal or differentiation program, respectively [173, 174]. The ASCL1 protein level is negatively regulated by an E3-ubiquitin ligase, HECT, UBA, and WWE domain-containing 1 (HUWE1), and inhibitor of DNA binding 4 (ID4), which reverses proliferating NSCs back into the quiescent stage [175, 176]. Mutations in the human *HUWE1* gene have been linked to X-linked ID [177, 178]. Genetic-screened homeobox 2 (GSX2), a homeodomain transcription factor, and tailless homolog (TLX), an orphan nuclear receptor, also play a critical role in promoting activation of subpopulation of V–SVZ NSCs [170, 179]. On the other hand, repressor element 1-silencing transcription factor (REST) and FoxO transcription factors are required for the maintenance of quiescent NSCs [171, 180–182].

Epigenetic regulations such as chromatin remodeling and histone modifications also play critical roles in regulating NSC behaviors by modulating gene expression in a long-lasting manner without altering genomic sequence [183]. B lymphoma Mo-MLV insertion region 1 homolog (BMI1), a core component of chromatin remodeling complex named polycomb repressive complex 1 (PRC1), controls mammalian NSC proliferation by repressing a cyclin-dependent kinase (CDK) inhibitor p16^INK4a^ [184]. In contrast, chromatin remodeling factor chromodomain-helicase-DNA–binding protein 7 (CHD7) maintains NSC quiescence through repressing the transcription of cyclins and CDKs and promoting the expression of Notch downstream effector *Hes5* [185]. Histone H2AX phosphorylation, following GABA_A_ receptor activation, limits V–SVZ NSC proliferation and self-renewal [186]. Histone deacetylase 3 (HDAC3) is important for NSC proliferation by regulating G2/M progression through stabilization of CDK1 [187]. Enhancer of zeste homolog 2 (EZH2), a subunit of PRC2, represses gene expression through H3K27 methylation and promotes NSC proliferation through regulating the PTEN/Akt/mTOR pathway [188].

### Molecular signatures, heterogeneity, and cell-cycle regulation of quiescent NSCs

Quiescence of stem cells has long been thought as a dormant state, passively waiting for activating signals [189]. Increasing evidence has changed this long-held paradigm and indicates that quiescence is actively maintained [83, 190]. This active maintenance of quiescence state in NSCs serves as a reserved pool of stem cells that can replace damaged stem cells for long-term somatic cell generation, insulating against risks of stem cell depletion and accumulation of tumorigenic mutations after multiple rounds of cell division [191]. Quiescent NSCs have unique molecular signatures that are distinct from those of proliferative NSCs. Transcriptomics with temporal analysis of molecular interplay during the transitioning of quiescence to activated stage in the SGZ and V–SVZ are revealed by using bulk and single-cell RNA sequencing (RNA-seq) [83, 99, 192, 193]. Quiescent NSCs in both niches have enriched expression of genes involved in cell–cell adhesion and cell–microenvironment interaction, suggesting that intrinsic and extrinsic signals are actively involved in maintaining stem cell quiescence [99, 193].

Using single-cell RNA-seq, quiescent NSCs in the V–SVZ are found to be heterogeneous and can be further subclassified into dormant state (qNSC1) and primed-quiescent state (qNSC2), with the latter being a transitory state in which genes involving protein synthesis and the cell cycle are up-regulated in preparation for subsequent reactivation [83]. A similar preactivation stage can be found in quiescent NSCs in the SGZ, in which protein translation capacity is up-regulated [193]. On the other hand, activated NSCs can be further subclassified into nondividing aNSC1 and dividing aNSC2, demonstrating that there exists a quiescent-activated continuum rather than a binary state [83]. From a metabolic perspective, the activation of quiescent NSCs involves transitioning from lipid metabolism, specifically glycolytic metabolism and fatty acid oxidation, to oxidative metabolism in the mitochondria [83, 193, 194]. While reactive oxygen species (ROS) are closely related to mitochondrial respiration, a study by Le Belle and colleagues showed that NADPH oxidase (NOX)-derived ROS enhance the shift of quiescent to proliferating NSCs as well as neurogenesis [195]. Whether mitochondrial-derived ROS play a role, if any, in enhancing this shift remains to be elucidated.

NSC heterogeneity has been the subject of immense study to categorize them into different matrices, e.g., morphology, site of origin, molecular signatures, etc., with important implications for understanding differential neurogenic capabilities among NSCs [196–198]. Within the SGZ, 2 variants of quiescent NSCs with distinct morphologies respond selectively to extrinsic stimulations, as physical exercise activates only radial NSCs, while seizure activates both radial and horizontal NSCs [199]. A recent study by Morizur and colleagues found that quiescent NSCs located in the V–SVZ display membrane receptors that are distinct from the activated NSCs and that niche signaling could be important in maintaining such a heterogenous population [99]. On the other hand, positional heterogeneity among NSCs within the V–SVZ is maintained even when they are grafted heterotopically or grown in vitro, implying that an inherent “memory” could be imprinted that may persist even when the external environment is changed [139].

It was widely believed that quiescent stem cells, including mammalian quiescent NSCs, arrest in the G_0_ stage. However, a recent study from Andrea Brand’s laboratory challenged this dogma by reporting that in the *Drosophila* VNC, the majority of quiescent NSCs (approximately 75%) arrest in the G_2_ stage, while the remaining approximately 25% of quiescent NSCs are in G_0_ [200]. An evolutionarily conserved pseudokinase, Tribbles, induces G_2_ NSCs to enter quiescence during late embryogenesis by targeting Cdc25^String^ for degradation [200]. During larval stages, Tribbles maintains G_2_ NSC quiescence by blocking Akt activation [200]. Activating the insulin pathway by overexpressing the activated form of Akt in NSCs represses the *tribbles* transcription, triggering NSC reactivation [200]. Compared with G_0_-arrested cells, G_2_ quiescent cells can reactivate more quickly in response to nutritional stimulus [200]. They also have the advantage of maintaining genomic integrity via high-fidelity homologous-recombination–mediated repair in response to DNA damage [200].

Whether quiescent NSCs arrest at the G_2_ or G_0_ stage is determined by a CDK inhibitor Dacapo (Dap)/p57^KIP2^ at the end of embryogenesis [201]. Dap directs NSCs to enter G_0_ quiescence, and loss of *dap* resulted in NSCs switching from G_0_ to G_2_ quiescence [201]. The G_2_/G_0_ quiescent NSCs have distinct spatial distribution, with G_0_ NSCs primarily occupying dorsal regions of the CNS and G_2_ NSCs primarily occupying ventral regions [201]. However, there is no bias for G_2_/G_0_ quiescent NSCs along the anterior–posterior axis [201]. These observations pose an interesting possibility that dorsal–ventral patterning factors may influence the choice between G_2_ and G_0_ quiescence. Indeed, the dorsal patterning transcription factor Muscle segment homeobox (Msh) directly binds to the enhancer sequence of *dap*, which is known to be sufficient for *dap* expression in the embryonic CNS, to promote G_0_ quiescence in a subset of dorsal NSCs [201, 202]. On the contrary, the ventral patterning factor ventral nervous system defective (Vnd), which is expressed in G2 quiescent NSCs that are located ventrally, does not have a role in promoting G_0_ quiescence [201].

The precise modulation of proliferation program in the activation of NSCs is important in maintaining the stem cell pool and generating differentiated neurons. One of the key differentiating hallmarks of quiescent and activated NSCs is the cell-cycle activity. In the mammalian brain, CDK-inhibitory proteins (CDKIs)/kinase inhibitory proteins (KIPs), i.e. p21^cip1/waf1^, p27^kip1^, and p57^kip2^, play the role of a molecular brake on the cell cycle during the G1 to S transition because their reduction leads to the activation of the proliferation program [203–205]. However, persistent abrogation of p21^cip1/waf1^ and p57^kip2^ ultimately leads to NSC exhaustion and impaired neurogenesis [203, 205]. As alluded earlier, the role of Dap, the *Drosophila* ortholog of p57^kip2^, in the spatial regulation of NSC quiescence demonstrates the conserved role of CDKIs in the negative regulation of NSC activation [206]. p16^INK4a^, another CDKI of the CDK-inhibitory protein/inhibitory protein of CDK4 (INK4) family—the expression of which increases in age—acts as a negative regulator of NSC activation in the V–SVZ only under the presence of neurogenic stimuli such as running [207]. Further studies on the remaining yet-to-be characterized CDKIs, i.e., p15^INK4b^, p18^INK4c^, and p19^INK4d^, in both *Drosophila* and mammalian systems will shed light on the possible interplay of these CDKIs in regulating NSC quiescence. Besides CDKIs, the tumor suppressor gene p53 also acts as an additional layer of regulation on NSC proliferation because the loss of p53 leads to radical activation of quiescent NSC in the V–SVZ [208]. Given that p53 is a key regulator of a variety of cellular processes, e.g., metabolism, senescence, etc., that are intimately linked to NSC activation, a holistic approach in studying how p53 might affect downstream activation genes is warranted [209].

## Conclusions and future perspectives

*Drosophila* represents an invaluable model system for in-depth dissection of molecular mechanisms underlying NSC quiescence and reactivation because of the conserved regulatory pathways shared with the mammalian brain and the availability of an arsenal of powerful genetic tools [13]. Future studies in mammalian systems on the conserved nature of the intrinsic regulators of NSC reactivation discovered in *Drosophila* will shed light on how these regulators might modulate stem cell behavior in a more complex system, with important implications in understanding neurological disorders and potential targets for therapeutic purposes. An emerging theme from the host of studies on molecular players governing NSC reactivation in *Drosophila* and mammalian system presented in this review is the complex, precise, and intricate balancing of quiescence and reactivation of NSCs within the neurogenic niche that allows them to respond to changes in the external environment and also the intrinsic development/aging clock in producing appropriate number of neurons while maintaining a stem cell pool for long-term neurogenesis. Thus, the dysregulation of these molecular players may result in neurodevelopmental diseases. A systems biology approach in understanding how NSCs reconcile and integrate the barrage of seemingly conflicting regulatory signals into a binary decision of quiescence or reactivation might prove useful in understanding the biology of NSC reactivation and the heterogeneity that exists within the NSC population.
